# Catheter Ablation Compared with Medical Therapy for Atrial Fibrillation with Heart Failure: A Systematic Review and Meta-analysis of Randomized Controlled Trials

**DOI:** 10.7150/ijms.52257

**Published:** 2021-01-21

**Authors:** Kuo-Li Pan, Yi-Ling Wu, Meng Lee, Bruce Ovbiagele

**Affiliations:** 1 Division of Cardiology, Department of internal medicine, Chang Gung Memorial Hospital, Chiayi, Taiwan.; 2 School of Medicine, Chang Gung University, Taoyuan, Taiwan.; 3 Heart Failure Center, Chang Gung Memorial Hospital, Chiayi branch, Taiwan.; 4 Institute of Population Health Sciences, National Health Research Institutes, Miaoli County, Taiwan.; 5 Department of Neurology, Chang Gung Memorial Hospital, Chiayi Branch, Taiwan.; 6 Department of Neurology, University of California, San Francisco, California, USA.; 7 San Francisco VA Healthcare System, San Francisco, California, USA.

**Keywords:** atrial fibrillation, heart failure, catheter ablation, medical therapy, mortality.

## Abstract

**Background:** The optimal strategy for patients with coexisting atrial fibrillation (AF) and heart failure (HF) was not settled. Our purpose was to conduct a systematic review and meta-analysis of randomized controlled trials to evaluate the effect of catheter ablation compared with medical therapy for AF on mortality, HF hospitalization, left ventricular (LV) function, and quality of life among patients with HF and AF.

**Materials and Methods:** We searched Pubmed (1966 to September 20, 2019), EMBASE (1966 to September 20, 2019), the Cochrane Central Register of Controlled Trials (CENTRAL), and ClinicalTrials.gov for randomized controlled trials with a comparison of catheter ablation for AF with medical therapy among patients with coexisting AF and HF. Risk ratio (RR) or mean difference (MD) with 95% confidence interval (CI) was used as a measure of the effect of catheter ablation versus medical therapy on endpoints. Our final analysis included 6 randomized control trials with 775 patients.

**Results:** Pooled results from the random-effects model showed that compared with medical therapy for AF, catheter ablation was associated with reduced all-cause mortality (RR 0.52, 95%Cl, 0.35 to 0.76) and HF hospitalization (RR 0.56, 95%Cl, 0.44 to 0.71), as well as increased LV ejection fraction (LVEF), distance walked in six minutes, and improvements in quality of life.

**Conclusions:** This updated meta-analysis showed that compared to medical therapy, catheter ablation for AF was associated with significant benefits in several key clinical and biomarker endpoints, including reductions in all-cause mortality and HF hospitalization.

## Introduction

Previous study showed that rhythm control with antiarrhythmic drugs does not reduce the rate of death among patients with coexisting HF and AF when compared to a rate-control strategy 1. Suboptimal efficacy in preventing AF, in addition to side effects from antiarrhythmic drugs, might offset the potential benefits of medical therapy by rhythm control. According to the 2020 European Society of Cardiology (ESC) Guidelines, catheter ablation for AF is a well-established treatment for symptomatic AF after failure of or intolerance drug therapy 2 and some studies suggested that catheter ablation of AF not only restores sinus rhythm, but also improves LV systolic function 3. However, the current European and American guidelines recommend a strategy using medical therapy as the first-line therapy in patients with coexisting AF and HF^2^, ^4^, possibly because a hard clinical endpoint, such as a reduction in mortality, has not been evaluated as a primary endpoint in published clinical trials.

Recently, a large randomized controlled trial showed that catheter ablation of AF, compared with medical treatment, significantly lowered the rate of mortality in patients with coexisting HF and AF 5. Based on these findings, in this study, we conducted an updated systematic review and meta-analysis of randomized controlled trials to evaluate the effects of catheter ablation versus medical therapy for AF on mortality, HF hospitalization, LV function, and quality of life, in patients with coexisting HF and AF.

## Methods

This study was performed in accordance with recommendations from the Preferred Reporting Items of Systematic Reviews and Meta-Analyses (PRISMA) statement 6.

### Data Sources and Searches

We searched Pubmed (1966 to September 20, 2019), EMBASE (1966 to September 20, 2019), the Cochrane Central Register of Controlled Trials (CENTRAL), and the clinical trial registry maintained at ClinicalTrials.gov with the terms: *“ablation or catheter”* and *“atrial fibrillation”* and *“heart failure”*. We restricted our search to human and clinical trials. There were no language restrictions. We also reviewed the Introduction and Discussion sections of retrieved trials and relevant review articles to identify additional trials. Two investigators (KLP and ML) independently conducted the literature search, screening of abstracts and selection of included trials.

### Study Selection

Criteria for inclusion of a study were as follows: (1) the study design was a randomized controlled trial; (2) the study population was comprised of patients with coexisting AF and HF; (3) the study included a comparison of catheter ablation for AF with medical therapy; (4) reported at least one of following endpoints in active and control groups: all-cause mortality, hospitalization for HF, remained in (or free from) AF, change in LVEF, 6-minute walk distance, and Minnesota Living with Heart Failure Questionnaire (MLHFQ).

### Data Abstraction

We abstracted data for baseline characteristics, including age, sex, duration of follow-up, and number of patients in each group. We abstracted data on endpoints from each trial, including number with all-cause mortality, hospitalization for HF, and remained in AF for catheter ablation versus medical therapy. We also abstracted information including increase in LVEF, increase in 6-minute walk distance, and decrease in MLHFQ in active and control group. Two investigators (KLP and ML) independently abstracted data from eligible studies. Any discrepant judgments were resolved by joint discussion that arrived at consensus.

### Data synthesis and analysis

The primary endpoint was all-cause mortality. Secondary endpoints were hospitalization for HF, remained in AF, change in LVEF, 6-minute walk distance, and MLHFQ. Risk ratio (RR) with 95% confidence interval (CI) was used to assess all-cause mortality, hospitalization for HF, and remained in AF, in the active group compared with control group. Mean difference with 95% CI was used to assess increase in LVEF, increase in 6-minute walk distance, and decrease in MLHFQ (i.e. lower score was considered as the better outcome) in the active group compared with control group. We pooled data from the random-effects model when two or more studies provided sufficient data (e.g. at least one event in either active or control group) for a given outcome. Heterogeneity was assessed by p value of χ^2^ and I^2^ statistics. Heterogeneity was considered if the χ^2^ test was significant (two-sided *p* < 0.05) or the I^2^ statistic was > 70 %. The fixed-effect and random-effects estimates of primary endpoint were compared to determine the influence of small-study effects on the results of our meta-analysis, as recommended by Cochrane Handbook for Systematic Reviews of Interventions 7. Also, meta-regression would be conducted if at least ten studies were included in this meta-analysis 7. Publication bias was assessed by funnel plot, which displayed standard error as the measure of sample size, and related risk as the measure of treatment effect on primary endpoint. We performed a sensitivity analysis to further explore the robustness of our results. To identify any studies that might have exerted a disproportionate influence on the summery treatment effect, we removed each individual trial from the meta-analysis, one at a time. The Newcastle-Ottawa Scale (NOS) using three pre-defined domains was used to evaluate the risk of bias of the included studies 8. Review Manager Software Package (RevMan version 5.3, The Cochrane Collaboration, London, UK) was used for meta-analysis.

## Results

**Figure 1:**
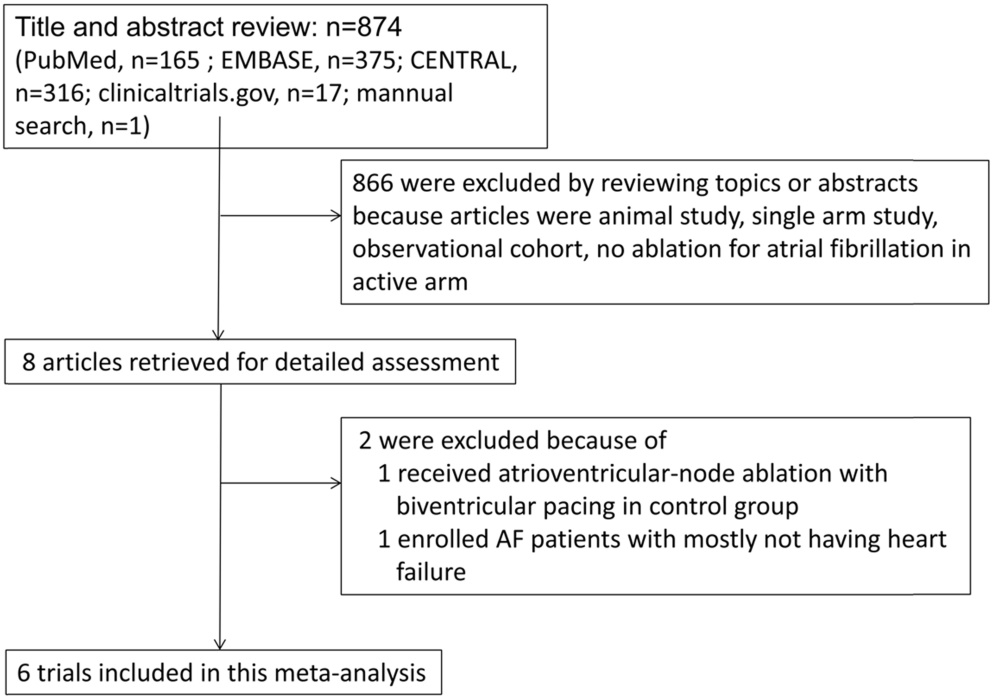
Flowchart of study selection

We identified eight full articles for detailed assessment, of which one was excluded based on participants in control group were receiving atrioventricular-node ablation with biventricular pacing 9, and one was excluded due to most participants not having HF 10. Our final analysis included six randomized control trials that included a total of 775 patients with systolic HF and AF (Figure 1) 5, 11-15.

Overall, 388 patients were randomly assigned to receive catheter ablation for AF and 387 patients were randomly assigned to receive medical therapy for rate and/or rhythm control. Most of the trials enrolled patients with persistent AF, but one large trial enrolled 33% patients with paroxysmal AF 5. Sample size ranged from 41 to 363 and 83% of participants were men. Four trials compared catheter ablation with rate control, one trial compared catheter ablation with amiodarone use, and one trial compared catheter ablation with medical therapy as guideline recommendation. Average age ranged from 57 to 64 years. Follow-up duration ranged from 6 to 38 months (Table 1).

Methods of measuring LVEF varied among included trials: two used radionuclide ventriculography 13, 15, two used transthoracic echocardiography 5, 12, and one used cardiac magnetic resonance 14. Most trials enrolled patients with New York Heart Association (NYHA) Functional class II-III clinically, with the exception of CASTLE-AF trial which enrolled a number of NYHA Functional class I or class IV patients 5. Average LVEF ranged from 18 to 35%. The underlying mechanisms for HF included both ischemic cardiomyopathy and non-ischemic cardiomyopathy (Table 2). Most patients received angiotensin-converting-enzyme inhibitor or angiotensin II receptor blockers and beta blocker treatment at baseline, while some patients also received digoxin and/or aldosterone antagonist (Supplemental Table 1). Assessment of risk of bias by Newcastle-Ottawa Scale was shown in Supplemental Table 2. The scores of Newcastle-Ottawa Scale were 8 or 9 out of 9 in included studies which suggested the high quality of these studies.

### Primary endpoint

**Figure 2:**
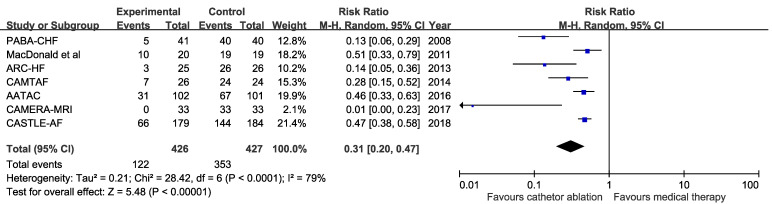
Risk ratio with 95% confidence interval of all-cause mortality (catheter ablation for atrial fibrillation vs medical therapy), by trial and pooled. M-H indicates Mantel-Haenszel methods.

For primary endpoint, two studies contained zero events in both ablation and medical groups and were excluded from the pooled analysis. Pooled results from random-effects model showed that catheter ablation for AF compared with medical therapy was associated with reduced all-cause mortality (Four trials; RR 0.52, 95%Cl, 0.35 to 0.76; number needed to treat=12) and there were no heterogeneity among trials (P for heterogeneity = 0.69, I^2^=0%) (Figure 2). Funnel plots showed no publication bias for all-cause mortality (Supplemental Figure 1). Results from sensitivity analyses showed no statistically significant difference from the overall pooled estimates. Analysis using fixed-effect model obtained similar results (Four trials; RR 0.52, 95%Cl, 0.36 to 0.77) and there were no heterogeneity among trials (P for heterogeneity = 0.69, I^2^=0%). The small study effect was not obvious because results were similar between random-effects and fixed-effect models. Meta-regression was not undertaken because of small number of trials included.

### Secondary endpoints

**Figure 3:**
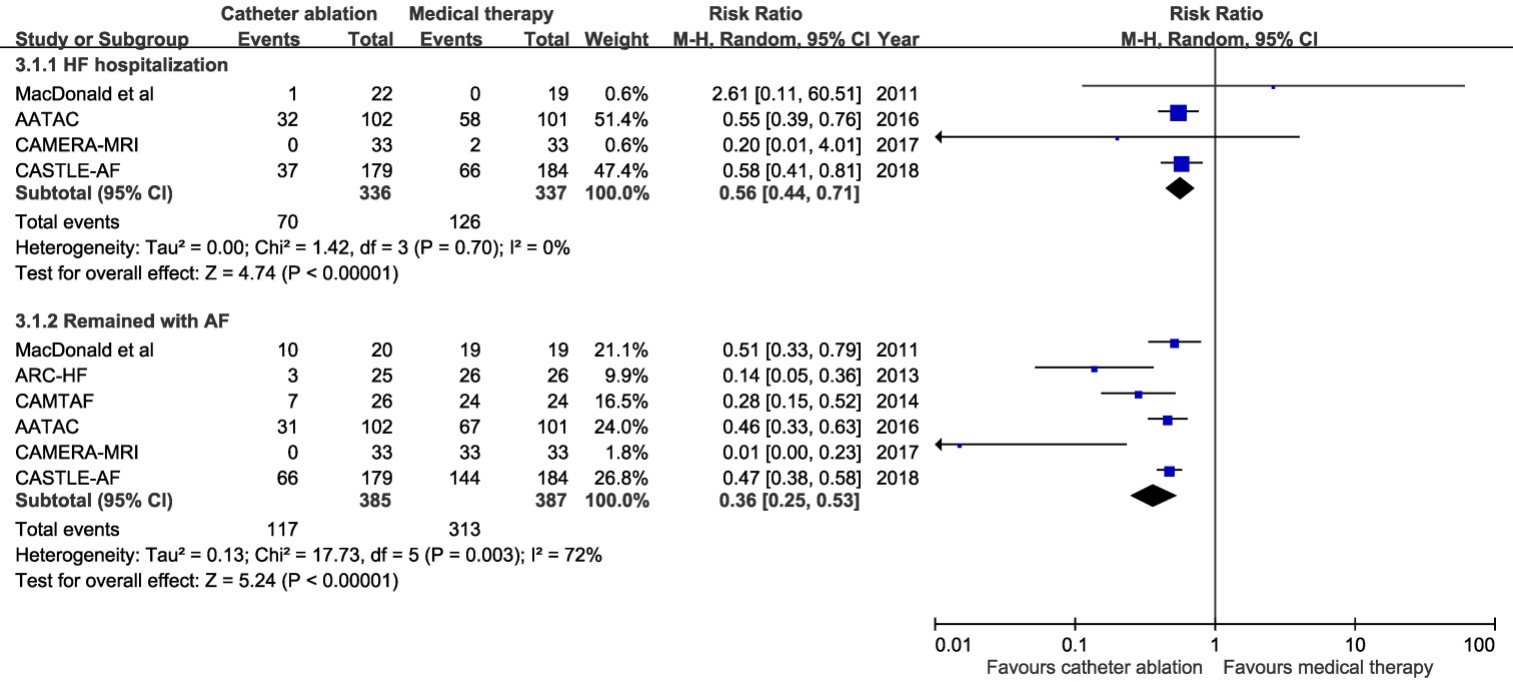
Risk ratio with 95% confidence interval of heart failure hospitalization and remained in atrial fibrillation (catheter ablation for atrial fibrillation vs medical therapy), by trial and pooled. HF: heart failure, AF: atrial fibrillation. M-H indicates Mantel-Haenszel methods.

Pooled results from the random-effects model showed that catheter ablation for AF compared with medical therapy was associated with reduced hospitalization for HF (4 trials; RR 0.56, 95%Cl, 0.44 to 0.71; number needed to treat =6) and patients who remained with AF (6 trials; RR 0.36, 95%Cl, 0.25 to 0.53; number needed to treat=2) (Figure 3). There were no substantial heterogeneity among trials.

**Figure 4:**
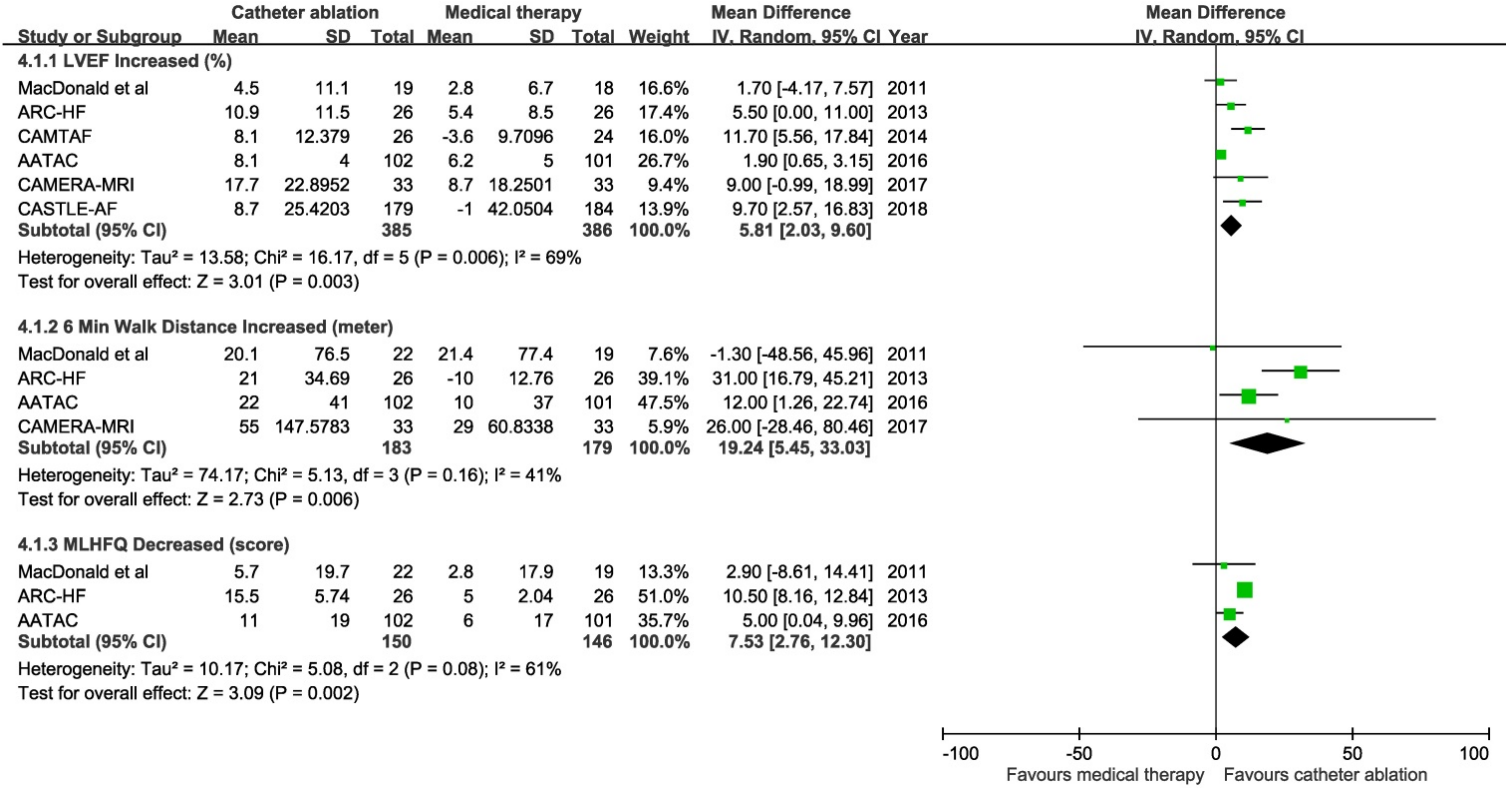
Mean difference with 95% confidence interval in left ventricular ejection fraction (LVEF), 6-minute walk distance, and Minnesota Living with Heart Failure Questionnaire (MLHFQ), by trial and pooled.

Pooled results from the random-effects model showed catheter ablation for AF compared with medical therapy was associated with increased LVEF from baseline (6 trials; mean difference 5.81%, 95% CI 2.03 to 9.60%), increased 6-minutes walking distance (4 trials; mean difference 19.24 meters, 95% CI 5.45 to 33.03 meters), and improved MLHFQ score (3 trials; mean difference 7.53, 95% CI 2.76 to 12.3) (Figure 4). There was heterogeneity among trials with an endpoint of LVEF change (P for heterogeneity = 0.006).

### Procedural Complications

Ablation related complications are summarized in Supplemental Table 3. Cardiac tamponade, pericardial effusion, and groin site bleeding that need blood transfusion were noted in some patients receiving catheter ablation in the included studies.

### Sensitivity Analysis with PABA-CHF Added to the Other Trials

Although PABA-CHF trial 9 was excluded due to participants in control group receiving atrioventricular-node ablation with biventricular pacing, we included this trial in the sensitivity analysis. No mortality or HF-related hospitalization was found in either group of PABA-CHF trial and had no impact in pooled analyses of relevant endpoints. Pooled results from the random-effects model showed that catheter ablation for AF was associated with reduced patients who remained with AF (Seven trials; RR 0.31, 95%Cl, 0.20 to 0.47; Supplemental Figure 2A) and increased LVEF from baseline (Seven trials; mean difference 6,46%, 95% CI 2.80 to 10.12%), increased 6-minutes walking distance (Five trials; mean difference 27.19 meters, 95% CI 8.14 to 46.24 meters), and improved MLHFQ score (Four trials; mean difference 10.78, 95% CI 3.57 to 17.99) compared with medical therapy (Supplemental Figure 2B).

## Discussion

In this meta-analysis comprised of six randomized controlled trials that enrolled over 700 patients with coexisting AF and HF, we found catheter ablation for AF was associated with 48% reduced risk for all-cause mortality, 44% reduced risk for HF-related hospitalization, and 59% reduced risk for AF compared with medical therapy. Additionally, catheter ablation for AF also showed significantly improved LV systolic function, increased the distance walked in six minutes, and improved the quality of life compared with medical therapy.

Evidence suggests that HF patients with AF have a worse prognosis than those whose sinus rhythm are maintained, and that the coexisting of AF is a risk factor for death 16-20. However, previously a large randomized controlled trial showed that a routine strategy of rhythm control using electrical cardioversion and amiodarone, did not reduce the rate of death from any cause or cardiovascular causes when compared with a rate-control strategy in patients with AF and HF, although about 75% of patients in the rhythm-control group were in sinus rhythm at repeated assessments during a three year follow-up period 1. Since a reduction in AF burden is both substantial in a previous trial 1 and our current meta-analysis but reduction of death was only seen in catheter ablation of AF, burden of AF might not be a major factor to determine mortality among these patients. On the other hand, higher LVEF was associated with a linear decrease in mortality in HF patients with LVEF ≤45% and sinus rhythm 21. We found that patients receiving catheter ablation for AF was associated with a 6% increase of LVEF, suggesting that the favorable outcomes reported with catheter ablation might be driven mostly by the improvement in LVEF.

AF and HF are both common heart diseases. Evidence suggests that AF is independently associated with an increased risk of sudden cardiac death 22. HF and low ejection fraction are predictors of sudden cardiac death in patients with AF 23, 24. AF significantly increases HF-related deaths and hospitalization in patients with HF 25. Also, in patients with HF, the risk of subsequent death is directly related to the duration and frequency of HF-related hospitalizations 26, 27. Therefore, effective interventions are needed to improve the prognoses of these high-risk patients. This study demonstrates that catheter ablation for AF compared with medical therapy reduced HF hospitalization, restored sinus rhythm, and improved LVEF, which may contribute to a lower mortality rate. Moreover, the control arm included both rate and rhythm control as the target strategy in two of the included trials 5, 11 which implied that medical therapy alone may no longer be the optimal choice for standard therapy.

This study has several limitations. First, a meta-analysis may be biased when the literature search fails to identify all relevant trials. To minimize these risks, we performed extensive search using multiple literature engines, and trial databases, and included recent review articles. Second, follow-up duration in most of the included trials were less than one year which is less likely to include mortality endpoint. Therefore, the results of primary endpoint, all-cause mortality were largely derived from two clinical trials. Still, both trials showed significantly decreased all-cause mortality rates for catheter ablation compared with medical therapy. Third, success of catheter ablation for AF relies largely on experienced operators, which should be considered when trying to generalize results from clinical trials to real-world practice.

## Conclusions

This updated meta-analysis showed that catheter ablation for AF compared with medical therapy was associated with risk reduction of all-cause mortality, hospitalization for HF, and AF, as well as increase of LVEF, exercise capacity, and life quality. In the presence of experienced medical operators, catheter ablation for AF might be considered as first-line therapy in patients with coexisting HF and AF.

## Supplementary Material

Supplementary figures and tables.

## Figures and Tables

**Table 1:** Baseline Characteristics of included trials

	Study population	Comparison	Sample size, n	% of men	Age, years	% of persistent AF	Follow-up duration, months
MacDonald et al. 2011, UK^14^	NYHA Functional class II-IV, LVEF <35% and persistent AF	Catheter ablation for AF/Medical therapy (rate control)	41	78	63	100	6
ARC-HF 2013, UK^16^	NYHA Functional class II-IV, LVEF ≤35% and persistent AF	Catheter ablation for AF/Medical therapy (rate control)	52	87	63	100	12
CAMTAF 2014, UK^13^	NYHA Functional class II-IV, LVEF <50% and persistent AF	Catheter ablation for AF/Medical therapy (rate control)	50	96	57	92	6
AATAC, 2016, European Countries and USA^12^	NYHA Functional class II-IV, LVEF ≤40% and persistent AF	Catheter ablation for AF/Medical therapy (amiodarone)	203	74	61	100	24
CAMERA-MRI 2017, Australia^15^	NYHA functional class ≥ II, persistent AF, LVEF ≤ 45% on baseline CMR	Catheter ablation for AF/Medical therapy (rate control)	66	91	61	100	6
CASTLE-AF 2018, European Countries and USA^6^	NYHA Functional class II-IV, LVEF ≤ 35% and recurrent AF	Catheter ablation for AF/in accordance with the guidelines)	363	86	64	67	38

AF: atrial fibrillation; LVEF: left ventricular ejection fraction; NYHA: New York Heart Association Class Trial name: AATAC: Ablation vs Amiodarone for Treatment of Atrial Fibrillation in Patients With Congestive Heart Failure and an Implanted ICD/CRTD; ARC-HF: A Randomised Trial to Assess Catheter Ablation Versus Rate Control in the Management of Persistent Atrial Fibrillation in Chronic Heart Failure; CAMTAF: Catheter Ablation Versus Medical Treatment of Atrial Fibrillation in Heart Failure; CAMERA-MRI: Catheter Ablation Versus Medical Rate control in Atrial Fibrillation and Systolic Dysfunction; CASTLE-AF: Catheter Ablation versus Standard Conventional Therapy in Patients with Left Ventricular Dysfunction and Atrial Fibrillation.

**Table 2:** Method of measuring LVEF and Characteristics of heart failure of included trials at baseline

	Method of measuring LVEF	NYHA I, %	NYHA II or III, %	NYHA IV, %	Average LVEF, %	% of ischemic heart failure	% of non-ischemic heart failure
MacDonald et al.^14^	Radionuclide ventriculography	0	100	0	18	49	51
ARC-HF^16^	Radionuclide ventriculography	0	100	0	23	33	67
CAMTAF^13^	Transthoracic echocardiography	0	100	0	33	26	74
AATAC^12^	Not mentioned	0	100	0	30	NA	NA
CAMERA-MRI^15^	Cardiac magnetic resonance	0	NA	NA	35	0	100
CASTLE-AF^6^	Transthoracic echocardiography	11	88	1	32	46	54

LVEF: left ventricular ejection fraction; NYHA: New York Heart Association Class Trial name: AATAC: Ablation vs Amiodarone for Treatment of Atrial Fibrillation in Patients With Congestive Heart Failure and an Implanted ICD/CRTD; ARC-HF: A Randomised Trial to Assess Catheter Ablation Versus Rate Control in the Management of Persistent Atrial Fibrillation in Chronic Heart Failure; CAMTAF: Catheter Ablation Versus Medical Treatment of Atrial Fibrillation in Heart Failure; CAMERA-MRI: Catheter Ablation Versus Medical Rate control in Atrial Fibrillation and Systolic Dysfunction; CASTLE-AF: Catheter Ablation versus Standard Conventional Therapy in Patients with Left Ventricular Dysfunction and Atrial Fibrillation CMR: cardiac magnetic resonance; NYHA: New York Heart Association; TTE: transthoracic echocardiography; SD: standard deviation
