# Supplementary material for: Catheter Ablation Compared with Medical Therapy for Atrial Fibrillation with Heart Failure: A Systematic Review and Meta-analysis of Randomized Controlled Trials
Source: Int J Med Sci. 2021 Jan 21;18(6):1325–31. doi: 10.7150/ijms.52257 (PMC7893556; doi:10.7150/ijms.52257)
Supplement: Supplementary file 1 — Supplementary figures and tables. [file ijmsv18p1325s1.pdf]

Supplemental Table 1 Percentage of medication used at baseline for included trials

	Digoxin	Aldosterone antagonist	Beta blocker	ACEI or ARB
MacDonald et al.[1]	51	32	88	95
ARC-HF[2]	54	37	92	98
CAMTAF[3]	NA	NA	100	100
AATAC[4]	NA	48	78	90
CAMERA-MRI[5]	NA	41	97	94
CASTLE-AF[6]	25	NA	94	93

NA: not available

Supplemental Table 2 The detailed assessment process of cohort studies/randomized clinical trials included into present meta-analyses based on the nine-star Newcastle-Ottawa Scale

	Selection 1)	Selection 2)	Selection 3)	Selection 4)	Comparability 1)	Outcome 1)	Outcome 2)	Outcome 3)	Score
MacDonald et al.[1]	a)	a)	b)	a)	a) b)	a)	b)	a)	8
ARC-HF[2]	a)	a)	b)	a)	a) b)	a)	a)	a)	9
CAMTAF[3]	a)	a)	b)	a)	a) b)	a)	b)	a)	8
AATAC[4]	a)	a)	b)	a)	a) b)	a)	a)	a)	9
CAMERA-MRI[5]	a)	a)	b)	a)	a) b)	a)	b)	a)	8
CASTLE-AF[6]	a)	a)	b)	a)	a) b)	a)	a)	a)	9

Supplemental Table 3 Complications of in a catheter ablation group

	Major complication	Minor complication
MacDonald et al.[1]	1 stroke<br>2 cardiac tamponade<br>3 worsening heart failure	NA
ARC-HF[2]	1 tamponade during ablation, requiring emergency pericardiocentesis<br>and a sternotomy to repair a perforation at the atrioventricular groove	1 groin hematoma, 1 chest infection 2 weeks post-ablation, and 1 patient with post-procedural pulmonary edema, which resolved within 24 h.
CAMTAF[3]	1 stroke<br>1 tamponade	NA
AATAC[4]	1 pericardial effusion that was conservatively managed with fresh-frozen plasma and protamine	2 groin hematoma
CAMERA-MRI[5]	1 groin site bleeding requiring blood transfusion<br>1 post-procedural pneumonia.	NA
CASTLE-AF[6]	3 pericardial effusion, and 1 of these patients required pericardiocentesis.<br>3 severe bleeding that required blood transfusion (with 2 bleeding episodes from femoral puncture sites and 1 pseudoaneurysm, which was corrected surgically)	1 asymptomatic pulmonary-vein stenosis

NA: not available

Supplemental Figure 1 Funnel plots of included trials

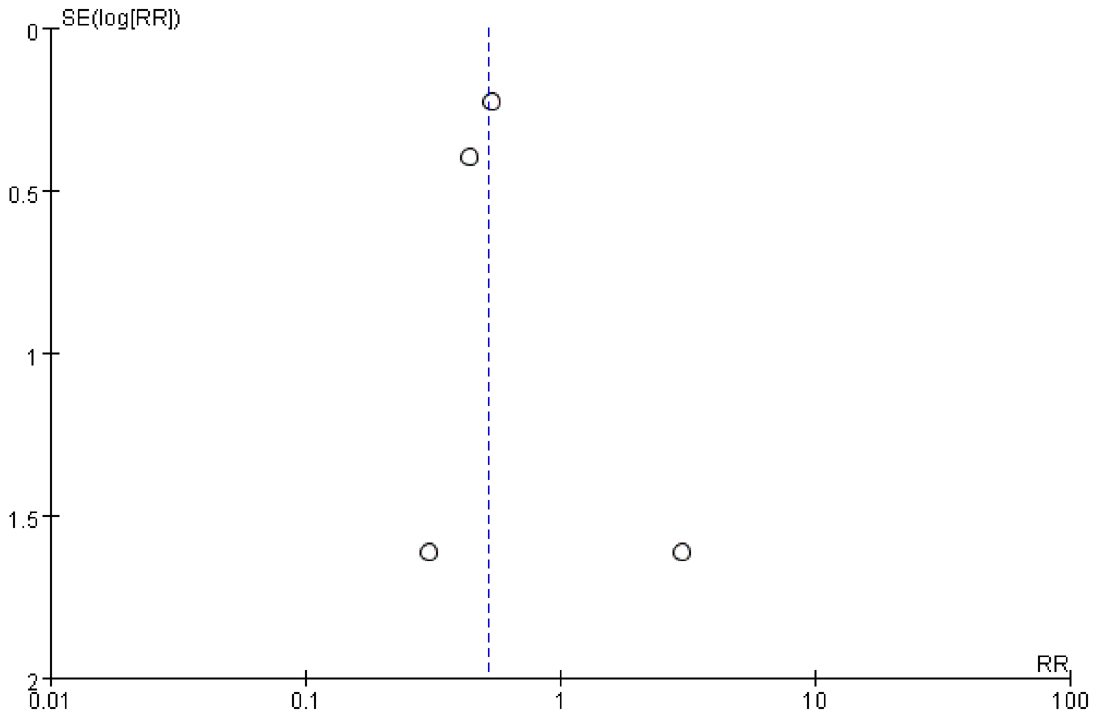

Supplemental Figure 2 Sensitivity Analysis with PABA-CHF[7] Added to the Other Trials (A) Risk ratio with 95% confidence interval of heart failure hospitalization and remained in atrial fibrillation (catheter ablation for atrial fibrillation vs medical therapy); (B) Mean difference with 95% confidence interval in left ventricular ejection fraction (LVEF), 6-minute walk distance, and Minnesota Living with Heart Failure Questionnaire (MLHFQ)

(A)

Study or Subgroup	Experimental Events	Experimental Total	Control Events	Control Total	Weight	Risk Ratio M-H, Random, 95% CI	Year
PABA-CHF	5	41	40	40	12.8%	0.13 [0.06, 0.29]	2008
MacDonald et al	10	20	19	19	18.2%	0.51 [0.33, 0.79]	2011
ARC-HF	3	25	26	26	10.2%	0.14 [0.05, 0.36]	2013
CAMTAF	7	26	24	24	15.3%	0.28 [0.15, 0.52]	2014
AATAC	31	102	67	101	19.9%	0.46 [0.33, 0.63]	2016
CAMERA-MRI	0	33	33	33	2.1%	0.01 [0.00, 0.23]	2017
CASTLE-AF	66	179	144	184	21.4%	0.47 [0.38, 0.58]	2018
**Total (95% CI)**		**426**		**427**	**100.0%**	**0.31 [0.20, 0.47]**	
Total events	122		353				

Heterogeneity: Tau² = 0.21; Chi² = 28.42, df = 6 (P < 0.0001); I² = 79%
Test for overall effect: Z = 5.48 (P < 0.00001)

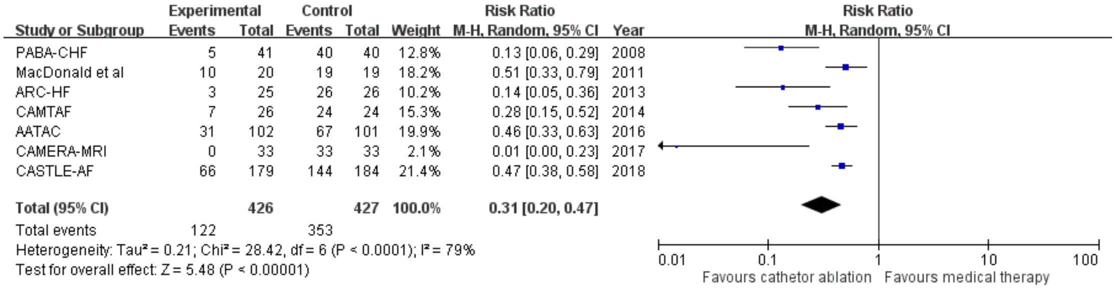

(B)

Study or Subgroup	Experimental Mean	Experimental SD	Experimental Total	Control Mean	Control SD	Control Total	Weight	Mean Difference IV, Random, 95% CI	Year
**2.2.1 LVEF increase (%)**									
PABA-CHF	8	9	41	-1	7	40	17.8%	9.00 [5.49, 12.51]	2008
MacDonald et al	4.5	11.1	19	2.8	6.7	18	13.7%	1.70 [-4.17, 7.57]	2011
ARC-HF	10.9	11.5	26	5.4	8.5	26	14.4%	5.50 [0.00, 11.00]	2013
CAMTAF	8.1	12.379	26	-3.6	9.7096	24	13.3%	11.70 [5.56, 17.84]	2014
AATAC	8.1	4	102	6.2	5	101	20.8%	1.90 [0.65, 3.15]	2016
CAMERA-MRI	17.7	22.8952	33	8.7	18.2501	33	8.2%	9.00 [-0.99, 18.99]	2017
CASTLE-AF	8.7	25.4203	179	-1	42.0504	184	11.8%	9.70 [2.57, 16.83]	2018
**Subtotal (95% CI)**			**426**			**426**	**100.0%**	**6.46 [2.80, 10.12]**	
Heterogeneity: Tau² = 16.39; Chi² = 27.43, df = 6 (P = 0.0001); I² = 78%									
Test for overall effect: Z = 3.46 (P = 0.0005)									
**2.2.2 6 Min walk Distance Increased (meter)**									
PABA-CHF	71	52	41	16	40	40	23.7%	55.00 [34.83, 75.17]	2008
MacDonald et al	20.1	76.5	22	21.4	77.4	19	10.8%	-1.30 [-48.56, 45.96]	2011
ARC-HF	21	34.69	26	-10	12.76	26	27.4%	31.00 [16.79, 45.21]	2013
AATAC	22	41	102	10	37	101	29.3%	12.00 [1.26, 22.74]	2016
CAMERA-MRI	55	147.5783	33	29	60.8338	33	8.9%	26.00 [-28.46, 80.46]	2017
**Subtotal (95% CI)**			**224**			**219**	**100.0%**	**27.19 [8.14, 46.24]**	
Heterogeneity: Tau² = 292.90; Chi² = 15.90, df = 4 (P = 0.003); I² = 75%									
Test for overall effect: Z = 2.80 (P = 0.005)									
**2.2.3 MLHFQ Decreased (score)**									
PABA-CHF	29	10	41	7	13	40	26.6%	22.00 [16.94, 27.06]	2008
MacDonald et al	5.7	19.7	22	2.8	17.9	19	17.2%	2.90 [-8.61, 14.41]	2011
ARC-HF	15.5	5.74	26	5	2.04	26	29.6%	10.50 [8.16, 12.84]	2013
AATAC	11	19	102	6	17	101	26.7%	5.00 [0.04, 9.96]	2016
**Subtotal (95% CI)**			**191**			**186**	**100.0%**	**10.78 [3.57, 17.99]**	
Heterogeneity: Tau² = 44.27; Chi² = 25.84, df = 3 (P < 0.0001); I² = 88%									
Test for overall effect: Z = 2.93 (P = 0.003)									

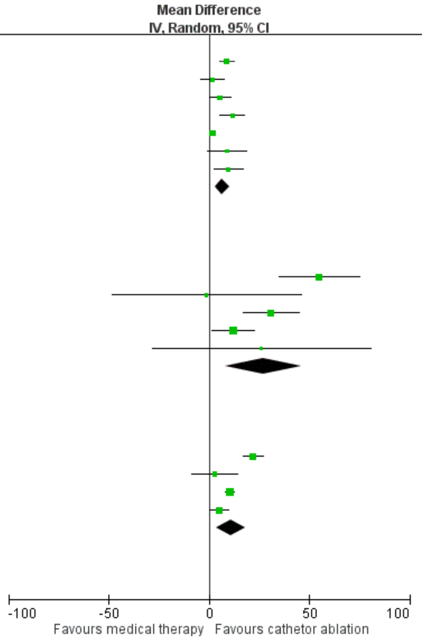

Test for subgroup differences: Chi² = 5.15, df = 2 (P = 0.08), I² = 61.1%

REFERENCE

1. MacDonald MR, Connelly DT, Hawkins NM, Steedman T, Payne J, Shaw M, Denvir M, Bhagra S, Small S, Martin W, McMurray JJ and Petrie MC. Radiofrequency ablation for persistent atrial fibrillation in patients with advanced heart failure and severe left ventricular systolic dysfunction: a randomised controlled trial. Heart. 2011;97:740-7.
2. Jones DG, Haldar SK, Hussain W, Sharma R, Francis DP, Rahman-Haley SL, McDonagh TA, Underwood SR, Markides V and Wong T. A randomized trial to assess catheter ablation versus rate control in the management of persistent atrial fibrillation in heart failure. Journal of the American College of Cardiology. 2013;61:1894-903.
3. Hunter RJ, Berriman TJ, Diab I, Kamdar R, Richmond L, Baker V, Goromonzi F, Sawhney V, Duncan E, Page SP, Ullah W, Unsworth B, Mayet J, Dhinoja M, Earley MJ, Sporton S and Schilling RJ. A randomized controlled trial of catheter ablation versus medical treatment of atrial fibrillation in heart failure (the CAMTAF trial). Circulation Arrhythmia and electrophysiology. 2014;7:31-8.
4. Di Biase L, Mohanty P, Mohanty S, Santangeli P, Trivedi C, Lakkireddy D, Reddy M, Jais P, Themistoclakis S, Dello Russo A, Casella M, Pelargonio G, Narducci ML, Schweikert R, Neuzil P, Sanchez J, Horton R, Beheiry S, Hongo R, Hao S, Rossillo A, Forleo G, Tondo C, Burkhardt JD, Haissaguerre M and Natale A. Ablation Versus Amiodarone for Treatment of Persistent Atrial Fibrillation in Patients With Congestive Heart Failure and an Implanted Device: Results From the AATAC Multicenter Randomized Trial. Circulation. 2016;133:1637-44.
5. Prabhu S, Taylor AJ, Costello BT, Kaye DM, McLellan AJA, Voskoboinik A, Sugumar H, Lockwood SM, Stokes MB, Pathik B, Nalliah CJ, Wong GR, Azzopardi SM, Gutman SJ, Lee G, Layland J, Mariani JA, Ling LH, Kalman JM and Kistler PM. Catheter Ablation Versus Medical Rate Control in Atrial Fibrillation and Systolic Dysfunction: The CAMERA-MRI Study. Journal of the American College of Cardiology. 2017;70:1949-1961.
6. Marrouche NF, Brachmann J, Andresen D, Siebels J, Boersma L, Jordaens L, Merkely B, Pokushalov E, Sanders P, Proff J, Schunkert H, Christ H, Vogt J, Bansch D and Investigators C-A. Catheter Ablation for Atrial Fibrillation with Heart Failure. The New England journal of medicine. 2018;378:417-427.
7. Khan MN, Jais P, Cummings J, Di Biase L, Sanders P, Martin DO, Kautzner J, Hao S, Themistoclakis S, Fanelli R, Potenza D, Massaro R, Wazni O, Schweikert R, Saliba W, Wang P, Al-Ahmad A, Beheiry S, Santarelli P, Starling RC, Dello Russo A, Pelargonio G, Brachmann J, Schibgilla V, Bonso A, Casella M, Raviele

A, Haissaguerre M, Natale A and Investigators P-C. Pulmonary-vein isolation for atrial fibrillation in patients with heart failure. The New England journal of medicine. 2008;359:1778-85.
